# Immunoglobulin G4 ‐related gastrointestinal disease associated with type 1 autoimmune pancreatitis: A case report

**DOI:** 10.1002/deo2.384

**Published:** 2024-05-07

**Authors:** Shingo Sato, Toyotaka Kasai, Hiroyuki Eto, Shikiko Okamoto, Ariki Nagashima, Rui Ushiyama, Reiri Shimazaki, Hiroshi Nitta, Motonori Arai, Hiroshi Ito

**Affiliations:** ^1^ Department of Gastroenterology Fukaya Red Cross Hospital Saitama Japan; ^2^ Department of Gastroenterology Kumagaya General Hospital Saitama Japan; ^3^ Department of Surgery Fukaya Red Cross Hospital Saitama Japan; ^4^ Department of Diagnostic Pathology Fukaya Red Cross Hospital Saitama Japan

**Keywords:** autoimmune pancreatitis, duodenal ulcer, endoscopy, gastrointestinal tract, immunoglobulin G4‐related disease

## Abstract

Immunoglobulin G4 (IgG4)‐related diseaseis a systemic inflammatory condition of unknown etiology characterized by increases in serum IgG4 and in the number of IgG4‐positive cells in affected tissues. One of the commonly involved locations is the pancreas; this condition is known as type 1 autoimmune pancreatitis (AIP). Type 1 AIP, which shows a biliary stricture in the intrapancreatic bile duct, can be misdiagnosed as a malignancy due to similar cholangiography findings and clinical presentation. In rare cases complicated by post‐bulbar duodenal ulcers, differentiating between type 1 AIP and malignancies is even more difficult. An 81‐year‐old male was referred to our hospital for the treatment of a pancreatic head mass and obstructive jaundice. Serological and radiological findings were consistent with both type 1 AIP and a malignancy. Gastroduodenoscopy revealed a post‐bulbar duodenal ulcer with endoscopic features that evoked malignant duodenal invasion. Although biopsies were negative for malignant cells, subsequent bleeding from the lesion suggested the progression of malignancy, which led to surgical resection. Pancreatoduodenectomy and pathological examination indicated that type 1 AIP was present. Simultaneously, the involvement of IgG4‐related disease in the ulcerative lesion was suggested. To our knowledge, this is the first reported case of type 1 AIP complicated by post‐bulbar duodenal ulcers, which was misdiagnosed as malignancy and considered an IgG4‐related gastrointestinal disease associated with type 1 AIP.

## INTRODUCTION

Immunoglobulin G4‐related disease (IgG4‐RD) involves various organs and structures such as the pancreas, biliary tract, salivary glands, retroperitoneum, aorta, kidney, orbit, and lung. However, its gastrointestinal tract involvement is rare, and this entity has not been fully clinicopathologically established.^1^ Type 1 autoimmune pancreatitis (type 1 AIP), in which a biliary stricture is found in the intrapancreatic bile duct, must be differentiated from pancreatic cancer, cholangiocarcinoma, and chronic pancreatitis. In most cases, type 1 AIP can be diagnosed based on clinical diagnostic criteria.^2^ Post‐bulbar duodenal ulcer (PBDU) is defined as a peptic ulcer that develops in the duodenum, anatomically more than 5 cm distal to the pyloric ring, and endoscopically distal to the superior duodenal flexure.^3^ A recent study demonstrated that PBDUs account for 20% of hemorrhagic duodenal ulcers.^3^ The etiology of PBDUs remains largely unresolved; however, there have been no reported cases of PBDU suggestive of IgG4‐related gastrointestinal disease (IgG4‐GID) nor associated with type 1 AIP. Additionally, evaluating whether a PBDU is benign or malignant based on its endoscopic and radiological features can be challenging, making the diagnosis of type 1 AIP even more difficult.

## CASE REPORT

An 81‐year‐old male was referred for treatment of obstructive jaundice. The patient had started experiencing generalized body weakness and anorexia a month prior, but no abnormal findings were noted on gastroduodenoscopy. When the patient developed jaundice, a low‐echoic mass was detected in the pancreatic head by abdominal ultrasonography.

**FIGURE 1:**
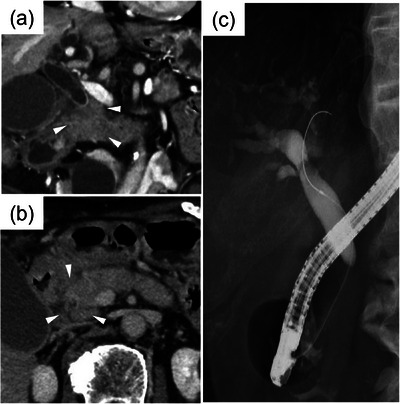
Computed tomography and endoscopic retrograde cholangiopancreatographyimages. Enhanced computed tomography showed stenosis in the common bile duct (arrowhead) by a poorly marginated mass around the pancreatic head (approximately 2 cm wide and 3 cm long), which also involved the second part of the duodenum (a, b). Endoscopic retrograde cholangiopancreatography showed a 5‐cm distal common bile duct stricture and dilated common and intrahepatic bile ducts (c).

**FIGURE 2:**
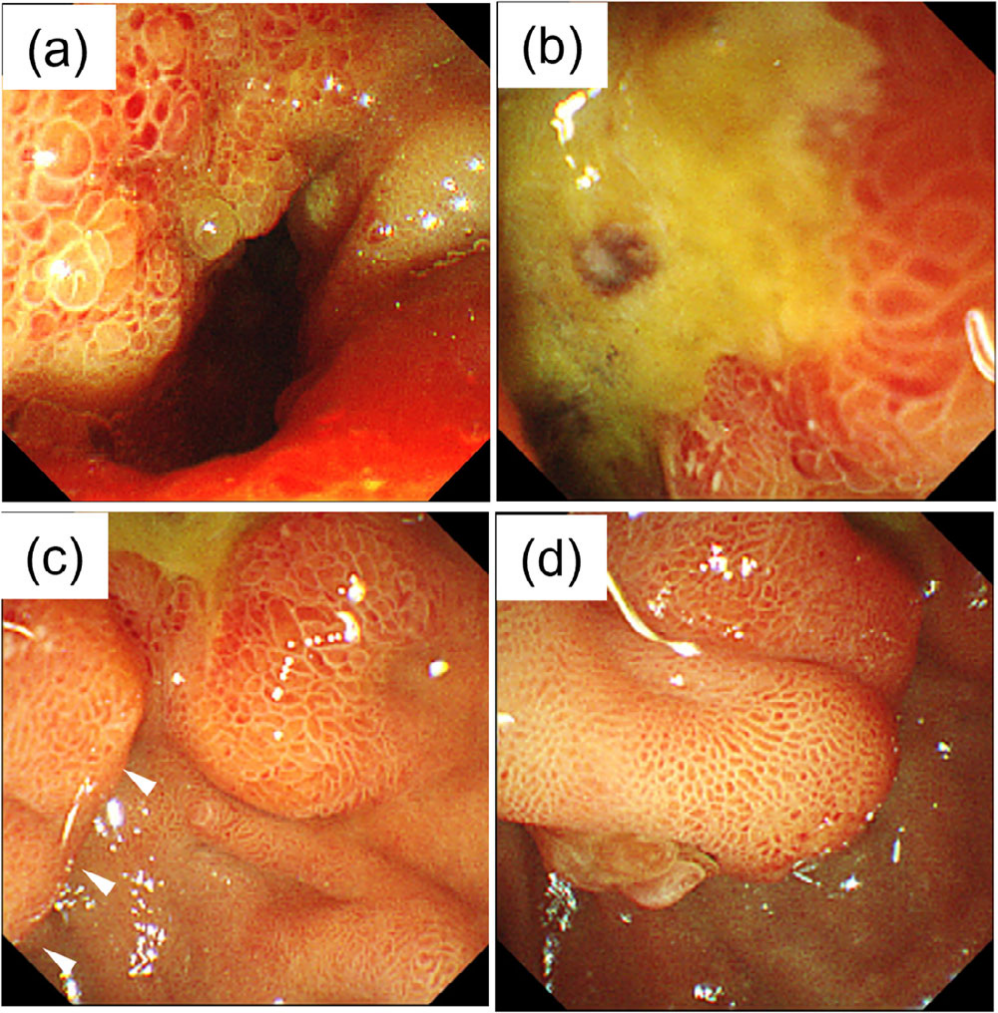
Duodenal images on the initial endoscopy. (a) A stenosed lumen with uneven and heterogeneously hypertrophied mucosa in the superior duodenal flexure. (b, c) A post‐bulbar duodenal ulcer with an irregular margin spread to the oral side of the papilla of Vater (arrowhead). (d) The oral protrusion is reddish and swollen.

At the initial visit, a laboratory workup revealed hyperbilirubinemia (total bilirubin, 19.4 mg/dL, and direct bilirubin 14.0 mg/dL) with elevated liver enzyme levels (aspartate aminotransferase, 100 U/L; alanine aminotransferase, 63 U/L). The cancer antigen 19‐9 level and serum IgG4 levels were elevated to 81 U/mL and 192 mg/dL, respectively. Enhanced computed tomography showed stenosis in the common bile duct (CBD) caused by a poorly marginated mass around the pancreatic head, which also involved the second part of the duodenum. Endoscopic retrograde cholangiography revealed a 5‐cm distal CBD stricture and dilated common and intrahepatic bile ducts (Figure 1a–c). A 7‐Fr plastic stent was inserted for biliary drainage. We found an ulcerative lesion with a stenosed lumen that had spread from the superior duodenal angle to the oral side of the papilla of Vater. The mucosa surrounding the ulcerative lesions was uneven and heterogeneously hypertrophied. The lesion margin was irregular, suggesting intraluminal invasion by the malignancy (Figure 2a–d).

No pathological evidence (i.e., brush cytology, bile cytology, or duodenal biopsy) supported a diagnosis of malignancy, but pancreatoduodenectomy (PD) was planned, considering the patient's current medical history and endoscopic findings.

**FIGURE 3:**
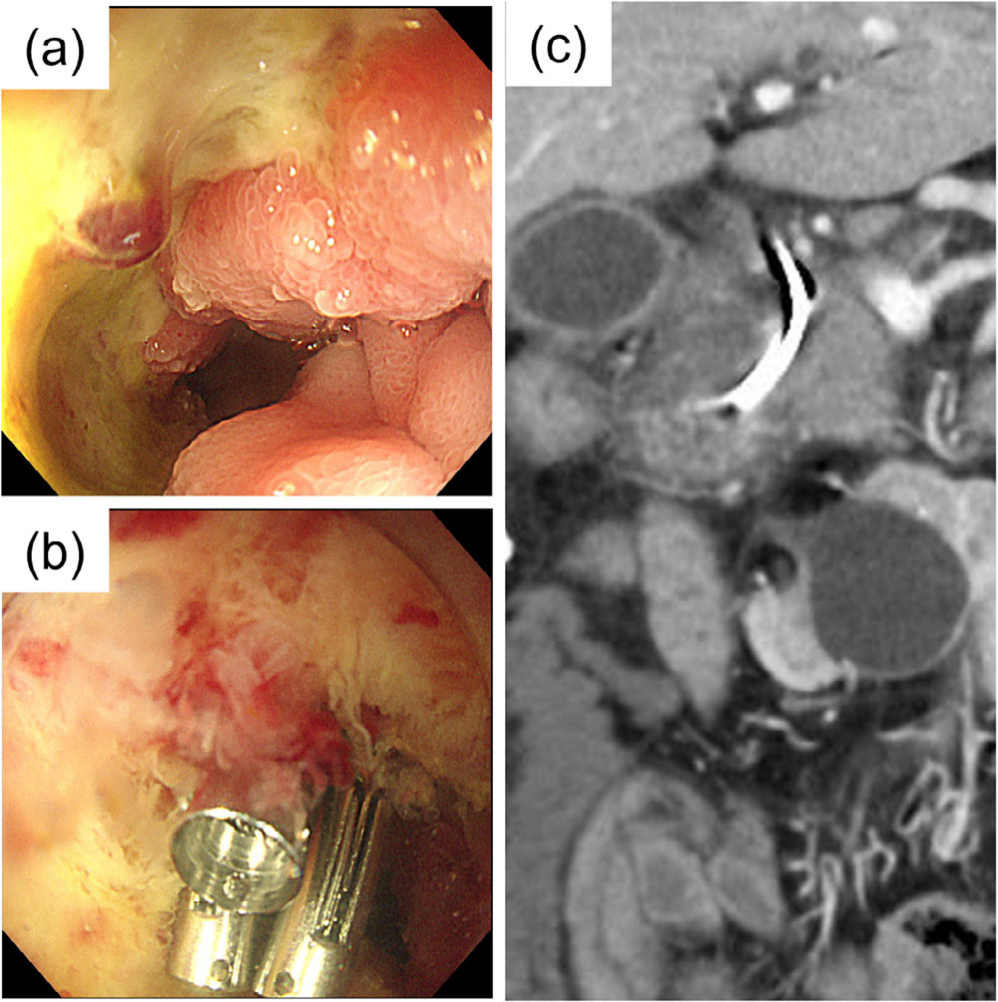
Endoscopic and computed tomography images of the duodenum showing a hemorrhagic event. (a) Post‐bulbar duodenal ulcer with an exposed vessel. (b) Endoscopic hemostat with three hemoclips (SureClip; Micro‐Tech Co., Ltd). (c) Thickened wall in the second part of the duodenum with the biliary stent. The presence of a thickened wall with internal heterogeneity was also misleading.

**FIGURE 4:**
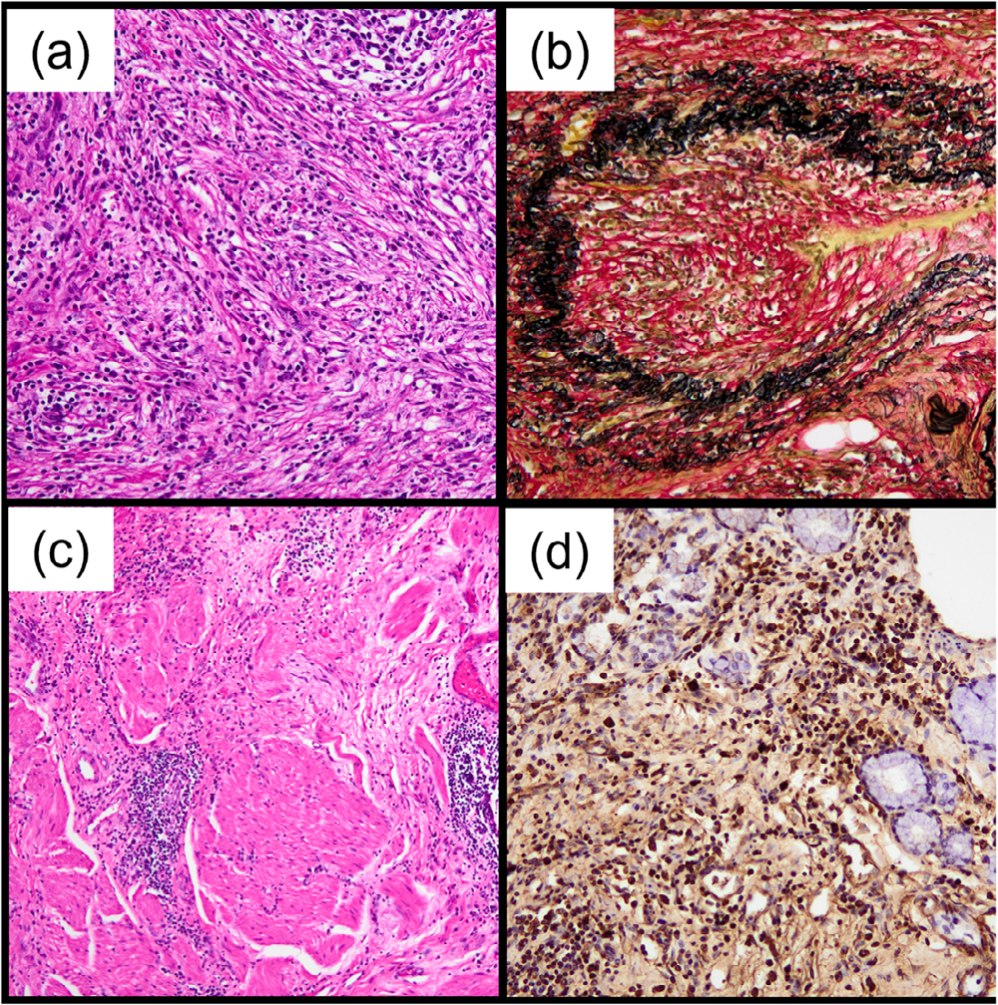
Histopathologic findings. (a, b) Storiform fibrosis and obliterative phlebitis observed in the pancreatic parenchyma ([a] hematoxylin and eosin staining, ×200; [b] Verhoeff's elastic staining, ×200). The number of immunoglobulin G4 (IgG4)‐positive plasma cells/high‐power field was 100 (not shown). (c) Lymphoplasmacytic infiltration within the muscularis propria, which resembled a “striated inflammatory lesion” ([c] duodenum; hematoxylin and eosin staining, ×100). (d) Lymphoplasmacytic infiltration in the mucosal lamina propria ([d] duodenum, IgG4 staining; ×200). The number of IgG4‐positive cells per high‐power field was over 50 and the IgG4/IgG‐positive cell ratio was 50% (not shown).

Two weeks after endoscopic biliary drainage, the patient was admitted to the emergency department with melena. Antacid treatment was discontinued due to diarrhea. Emergency gastroduodenoscopy revealed an advanced duodenal ulcer with an exposed vessel. Endoscopic hemostasis was performed using the clipping technique (Figure 3a–c). This hemorrhagic event was interpreted as an aggravation of the intraluminal invasion by malignancy. PD was performed after the patient recovered from bleeding. Endoscopic ultrasonography was an option, but our preoperative diagnosis was so biased by the PBDU endoscopic findings that it was not planned. Histopathological examination of the resected specimen revealed lymphoplasmacytic infiltration of IgG4‐positive plasma cells in the pancreatic parenchyma and intrapancreatic CBD. The patient was finally diagnosed with type 1 AIP (Figure 4a,b). Abundant IgG4‐positive cells were also found in the inflamed lesion of the duodenal wall (Figure 4c,d), suggesting IgG4‐RD involvement. The patient is being followed every 3 months on our outpatient using serum biochemical tests and serum IgG4 levels. Corticosteroid treatment was refused due to concerns about the side effects associated with long‐term administration. No sign of relapse has been observed so far.

## DISCUSSION

IgG4‐RD is a systemic inflammatory disorder characterized by the infiltration of IgG4‐positive cells into affected organs. Although the pancreas and hepatobiliary system are commonly affected, IgG4‐RD involvement in the gastrointestinal tract is rare.^1^ To our knowledge, there are 19 reported cases in the literature that present gastrointestinal obstruction and gastroduodenal ulcer.^1^, ^4^, ^5^, ^6^ Among these 19 cases, there was only one case of multiple duodenal ulcers leading to duodenal obstruction.^5^ Although these ulcers have been described as IgG4‐RD, no specific relationship with type 1 AIP has been noted.

Our patient presented with type 1 AIP complicated by PBDU. The etiology of PBDUs remains unclear; however, they seem unrelated to *Helicobacter pylori* infection, nonsteroidal anti‐inflammatory drug intake, and psychological stress, which are well‐recognized causes of ordinary peptic ulcers.^3^, ^7^ In our case, the histopathological examination of the resected specimen suggested IgG4‐RD involvement in the formation of the PBDU. Notohara et al.^1^ defined cases with IgG4‐positive cells > 50/high‐power field (HPF) and IgG4/IgG‐positive cell ratio > 40% observed in the three foci with the most abundant IgG4‐positive cells as “possible IgG4‐GID” and as “highly suggestive of IgG4‐GID”, if storiform fibrosis, obliterative phlebitis and/or perineural inflammation was further present in cases of “possible IgG4‐GID”. Our case had over 50 IgG4‐positive cells in the resected specimen of the duodenal wall per HPF and the IgG4/IgG‐positive cell ratio was 50%. A typical “bottom‐heavy plasmacytosis in the mucosal lamina propria”,^1^ which was distinct from *H. pylori*‐associated gastritis, was not found, but what resembled a “striated inflammatory lesion”^1^ was also found within the muscularis propria. Given the positive result of anti‐*H. pylori* IgG (Denka kit; Denka Seiken Co., Ltd) of 24 U/ml, the patient might have had an *H. pylori* infection. However, the pathology of the duodenal ulcer in this case still suggested the presence of IgG4‐RD rather than *H. pylori* infection.

Endoscopic images can be useful for diagnosis in cases where no sufficient tissue is obtained to rule out malignancy. Therefore, it is important to elucidate the differences between malignant and benign endoscopic characteristics of ulcers. The features of gastric ulcers with malignant potential include dirty bases, elevated ulcer borders, and irregular ulcer borders. Ulcers > 3 cm in size are also more likely to be malignant.^8^ For duodenal ulcers, only a few reports have described what endoscopic images are useful to determine the malignant potential.^9^, ^10^ Conversely, the potential of imaging to ascertain benignity in daily practice has been established. Benign lesions have a regular outline and sometimes appear as a punched‐out lesion on the duodenal wall. In the present case, the mucosa surrounding the ulcerative lesion was uneven and heterogeneously hypertrophic, with irregular and rolled margins (Figure 2a–c). It was more related to lymphoma than pancreatic cancer^9^, ^10^; either way, it suggested the presence of malignancy.

Our preoperative diagnosis was substantially affected by the endoscopic findings of PBDU. Without any pathological evidence, clinicians must apply a diagnostic procedure other than endoscopy to achieve more accurate differentiation. A possible alternative is treatment with steroids and proton pump inhibitors.^2^, ^4^ Another possible method for differentiation is pancreatic biopsy. In our case, the PBDU advancement with bleeding caused us to refrain from steroid treatment and serial endoscopic observation. However, endoscopic ultrasound‐fine‐needle aspiration/biopsy was feasible if performed from the stomach. In AIP, the detection rates of lymphoplasmacytic infiltration, obliterative phlebitis, and storiform fibrosis have been reported to be 84%–100%, 24%–43.6%, and 56%–72%, respectively.^2^ We should have tried this procedure at least once, considering the burden of PD on this elderly patient. This was one of the important lessons brought by this case.

In conclusion, we report the first case of type 1 AIP complicated by PBDU which is suggestive of IgG4‐GID, reminding clinicians that type 1 AIP mimics malignant tumors. There is still room for improvement in the diagnostic approaches to prevent unnecessary resections.

## CONFLICT OF INTEREST STATEMENT

None.
